# Abdominal aneurysm sac thrombus CT density and volume after EVAR: which association with underlying endoleak?

**DOI:** 10.1186/s41747-024-00489-3

**Published:** 2024-08-01

**Authors:** Matthias Lembrechts, Lucas Desauw, Walter Coudyzer, Annouschka Laenen, Inge Fourneau, Geert Maleux

**Affiliations:** 1 Department of Radiology, University Hospital Leuven, Leuven, Belgium; 2 Department of Imaging and Pathology, KU Leuven, Leuven, Belgium; 3 Department of Biostatistics and Statistical Bioinformatics, University Hospitals Leuven, Leuven, Belgium; 4 Department of Vascular Surgery, University Hospitals Leuven, Leuven, Belgium; 5 Department of Cardiovascular Sciences, University Hospitals Leuven, Leuven, Belgium

**Keywords:** Abdominal endovascular procedures, Aortic aneurysm, Endoleak, Endovascular aneurysm repair, Tomography (x-ray computed)

## Abstract

**Background:**

Our aim was to analyse abdominal aneurysm sac thrombus density and volume on computed tomography (CT) after endovascular aneurysm repair (EVAR).

**Methods:**

Patients who underwent EVAR between January 2005 and December 2010 and had at least four follow-up CT exams available over the first five years of follow-up were included in this retrospective single-centre study. Thrombus density and aneurysm sac volume were calculated on unenhanced CT scans. Linear mixed models were used for data analysis.

**Results:**

Out of 82 patients, 44 (54%) had an endoleak on post-EVAR contrast-enhanced CT. Thrombus density significantly increased over time in both the endoleak and non-endoleak groups, with a slope of 0.159 UH/month (95% confidence interval [CI] 0.115–0.202), *p* < 0.0001) and 0.052 UH/month (95% CI 0.002–0.102, *p* = 0.041). In patients without endoleak, a significant decrease in aneurysm sac volume was identified over time (slope -0.891 cc/month, 95% CI -1.200 to -0.581); *p* < 0.001) compared to patients with endoleak (slope 0.284 cc/month, 95% CI -0.031 to 0.523, *p* = 0.082). The association between thrombus density and aneurysm sac volume was positive in the endoleak group (slope 1.543 UH/cc, 95% CI 0.948–2.138, *p* < 0.001) and negative in the non-endoleak group (slope -1.450 UH/cc, 95% CI -2.326 to -0.574, *p* = 0.001).

**Conclusion:**

We observed a progressive increase in thrombus density of the aneurysm sac after EVAR in patients with and without endoleak, more pronounced in patients with endoleak. The association between aneurysm volume and thrombus density was positive in patients with and negative in those without endoleak.

**Relevance statement:**

A progressive increase in thrombus density and volume of abdominal aortic aneurysm sac on unenhanced CT might suggest underlying endoleak lately after EVAR.

**Key Points:**

- Thrombus density of the aneurysm sac after EVAR increased over time.
- Progressive increase in thrombus density was significantly associated to the underlying endoleak.
- The association between aneurysm volume and thrombus density was positive in patients with and negative in those without endoleak.

**Graphical Abstract:**

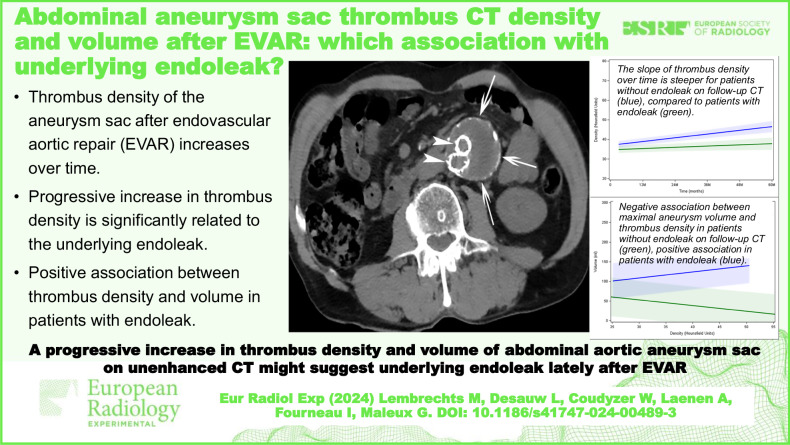

## Background

Endovascular aneurysm repair (EVAR) has become the standard treatment of care for patients with an infrarenal aortic aneurysm [1]. However, endoleaks are still the Achilles’ heel of EVAR and radiological follow-up mainly focuses on the detection of endoleaks. Endoleak can be considered as blood leakage in the excluded aneurysm sac and results in pressurisation of the sac with subsequent risk for rupture [2, 3].

Contrast-enhanced computed tomography (CE-CT) is considered the modality of choice for identifying endoleaks during follow-up [4]. However, it has several disadvantages, including the need for iodinated contrast agent administration and repeated radiation exposure [4, 5]. In order to counteract these disadvantages, several researchers have analysed the potential values of alternative imaging techniques to evaluate the aneurysm sac after EVAR, in particular, the presence of endoleaks: duplex- or contrast-enhanced ultrasound as well as magnetic resonance imaging (MRI) has been proposed. However, these alternative techniques have also disadvantages. Ultrasound is operator-dependent and the retroperitoneal structures or entire aorta are not always accessible for ultrasound examination [6, 7]. In some institutions MRI is not always available for aortic follow-up imaging; in addition, EVAR, performed with endografts with a stainless-steel skeleton or with ancillary stainless-steel coil-embolisation of the internal iliac artery or inferior mesenteric artery, may be limited on MRI by massive susceptibility artefacts [7, 8].

Therefore, we conducted an imaging study to evaluate the potential value of unenhanced CT datasets for the detection of endoleaks during follow-up after EVAR based on thrombus density and aneurysm sac volume measurements.

## Methods

### Patient selection

This is a retrospective single-centre study including consecutive patients who underwent EVAR at the authors’ institution between January 2005 and December 2010. In addition, at least four follow-up CT studies within five years after EVAR were also considered as a strict inclusion criterion; patients with less than four follow-up CT studies or patients with ultrasound follow-up, were excluded from the analysis. No precise patient sample size could be calculated for this pilot study related to the unavailability of studies dealing with the same topic. The study was approved by the Institutional Ethics Committee (S66615).

### CT protocol

Follow-up imaging after EVAR was performed with triphasic CT protocol, including a native scan, followed by an arterial and a late venous phase. Scan delay for the early, arterial phase was individualised using a bolus-tracking technique after intravenous injection of 100 mL of nonionic iodinated contrast medium Iomeprol (Iomeron 350, Bracco Imaging, Collaretto Giacosa, Italy) and triggering on the thoracoabdominal aorta (intra-arterial enhancement of 120 HU); no individual adjustment of the amount of contrast medium to the patient’s body weight was made. The injection rate was fixed at 4 cc/s for all patients. The late venous phase was performed 90 s after intravenous contrast medium administration; all CT studies were performed on a Sensation 64 scanner (Siemens Healthineers, Forchheim, Germany). The imaging parameters were: detector collimation 128 × 0.6 mm; helical pitch 1.2; gantry rotation time 0.5 s; tube voltage for normal size patient 120 kV; and planned tube current-time product per rotation for normal patient size, 210 mAs. Automatic tube current modulation adjusted the tube loads to different patient sizes. Reconstructed images of 3-mm and 1-mm thickness were available in axial, reformation; coronal and sagittal reformatted images were available in 3-mm thickness for analysis.

### Image analysis

Endoleaks were identified in early and late contrast-enhanced phases by a senior vascular radiologist (GM) with 30 years of experience in aortic CT imaging. After intravenous injection of iodine contrast agent, there was a clear enhancement of (parts of) the excluded aneurysm sac related to proximal/distal perigraft flow (type I endoleak), inversed flow in aortic side branches (type II endoleak) or direct flow into the sac related to a graft fabric tear or to graft component disconnection (type III endoleak).

**Fig. 1:**
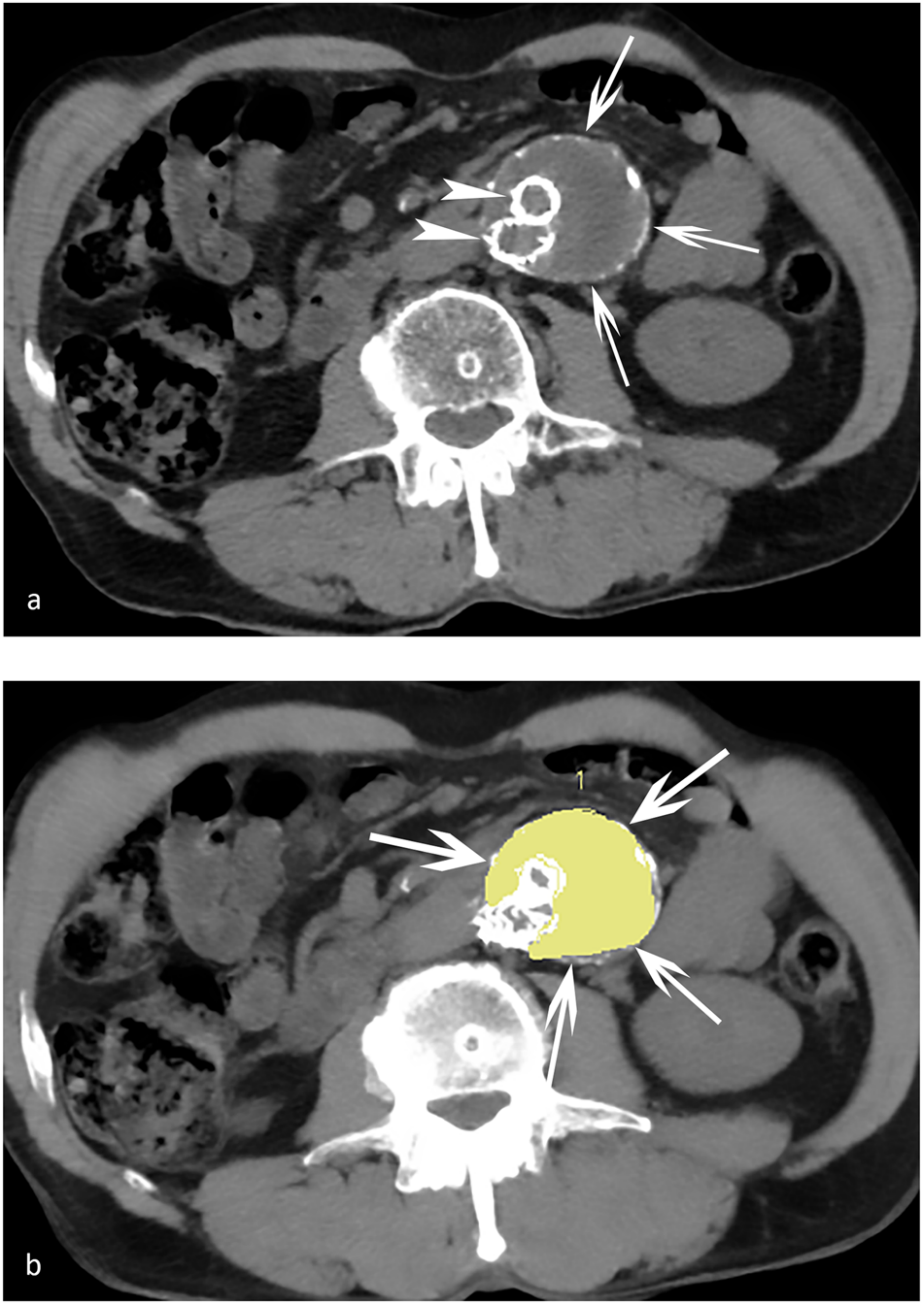
Follow-up unenhanced computed tomography one year after endovascular aortic repair. **a** The endograft limbs (arrowheads) within the excluded aneurysm sac (arrows) are visible. **b** Sac thrombus density was calculated after segmenting the sac thrombus (yellow-coloured area, arrows) on every axial computed tomography slice

Thrombus density and aneurysm volume were independently measured on a dedicated workstation (SyngoVia, Siemens Healthineers, Forchheim, Germany) by two radiologists in training (M.L. and L.D.) with one and five years of experience in diagnostic radiology, including CT, respectively. Mean thrombus density was calculated in HU, aortic aneurysm volume in cm^3^ and length of the aortic aneurysm in cm on the Siemens Volume Viewer (Siemens Healthineers, Forchheim, Germany). Thrombus density calculation was performed on the anonymised unenhanced CT series after manual segmentation of the aneurysm sac thrombus on all axial, 3-mm-reconstructed slices, thereby excluding the endograft skeleton and perfused lumen as well as the calcified aortic wall (Fig. 1). All densities within the range of -30 and 125 HU were included; outliers were excluded to reduce the influence of mainly high densities, including aortic wall calcifications and metallic skeleton of the endograft. Thrombus density, aneurysm volume and length were calculated on standard time points during follow-up and included 3, 6, 9, 12 and 18 months after EVAR; further follow-up included 2, 3, 4 and 5 years of follow-up after EVAR.

Reproducibility was tested on the first 10 patients included in the study.

### Statistical analysis

Linear mixed models were used for data analysis including random effects to account for the longitudinal data structure. To investigate the evolution of thrombus density or aneurysm volume over time, the fixed effects models included time, presence/absence of endoleak, and the interaction between time and endoleak. To investigate the association between thrombus density and aneurysm volume, the model included aneurysm volume as the response variable, and thrombus density, endoleak, and the interaction between thrombus density and endoleak as the fixed effects.

The intraclass correlation coefficient was calculated as a measure of intraobserver and interobserver variability.

All tests were two-sided and performed at a 5% significant level. Analyses have been performed using SAS software (version 9.4 of the SAS System for Windows, Cary, NY, USA) by one coauthor who is a biostatistician (A.L.).

## Results

**Fig. 2:**
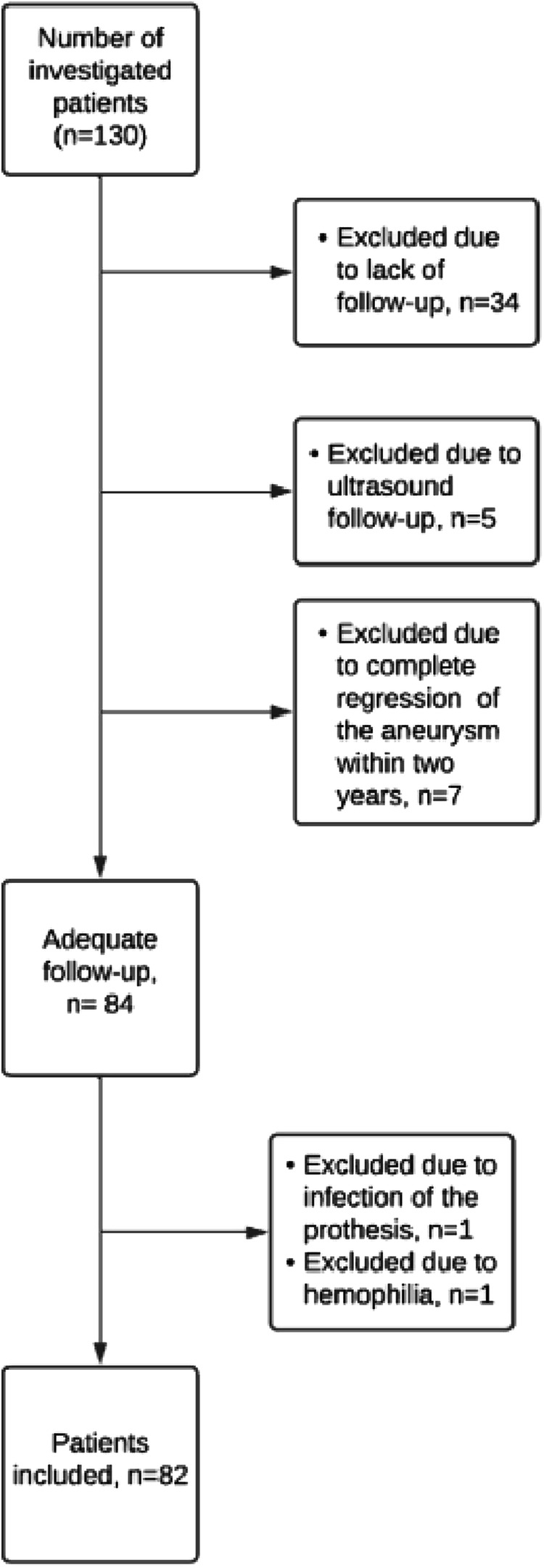
The study flow chart shows that most of the patient exclusions were related to lack of imaging follow-up (*n* = 34) or to the technical impossibility of segmenting the thrombus due to complete regression of the aneurysm sac within two years of follow-up (*n* = 7)

A total of 82 patients (6 women, 76 men; mean age 73 years, ranging from 58 to 88 years) were included in the study as summarised in the study flow chart (Fig. 2). In 44 of 82 patients (54%) an endoleak was identified on follow-up contrast-enhanced CT. Out of these 44 patients, 36 (82%) had a primary type II endoleak and 8 (18%) a secondary type I endoleak, including four proximal type I and four distal type Ib endoleak. The distal type Ib endoleaks were managed with an additional stent-graft limb, extending into the external iliac artery after proximal coil-embolisation of the internal iliac artery (*n* = 2); by open repair (*n* = 1) or by conservative management related to severe comorbidities (*n* = 1). The proximal type Ia endoleaks were managed with a proximal aortic cuff in three patients and by conservative management related to severe comorbidities in one patient.

**Fig. 3:**
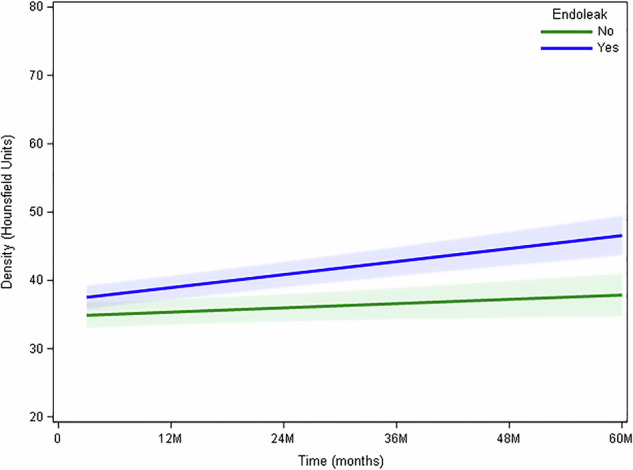
The slope of the curve for the relationship between thrombus density is steeper for patients without endoleak on follow-up CT scans (blue curve, *p* < 0.001) compared to patients with endoleak on follow-up CT scans (green curve, *p* = 0.040). M, Months

A total of 479 CT scans were analysed for density, volume and length measurements (mean of 5.8 follow-up CT scans per patient); the results of these measurements are summarised in Table 1. Patients with an endoleak on follow-up CT had a significant increase in thrombus density over time over a slope of 0.159 UH/month (95% confidence interval [CI] (0.115–0.202), *p* < 0.0001). In patients without endoleak, there was a significant increase in thrombus density over time as well, however, the slope of the curve is clearly more leveled at 0.05 UH/month (95% CI (0.002–0.102), *p* = 0.040) (Fig. 3).

**Table 1:** Aneurysm volume, length and density during follow-up computed tomography

Variable	Statistic	3 months	6 months	9 months	12 months	18 months
Volume (cm^3^)	Number	50	48	13	71	33
	Mean	116.1	89.5	79.4	90.6	69.9
	SD	177.1	68.1	41.2	151.0	423.0
	Median	73.2	65.8	63.5	57.8	57.2
	IQR	(58.1–127.7)	(44.8–114.9)	(54.7–101.0)	(37.2–98.9)	(39.4–94.6)
	Range	(9.4–1283.2)	(15.6–327.9)	(6.2–137.9)	(1.3–1236.4)	(13.3–170.5)
Heigth (cm)	N	50	48	13	71	33
	Mean	7.5	6.9	7.0	6.6	6.38
	SD	2.4	2.2	2.0	2.3	2.3
	Median	7.3	6.5	7.0	6.0	6.0
	IQR	(5.7–9.0)	(5.4–8.0)	(6.0–8.4)	(5.4–7.8)	(5.0–7.2)
	Range	(2.4–14.5)	(3.5–12.5)	(2.7–10.0)	(1.5–15.0)	(3.5–13.2)
Density (HU)	Number	50	48	13	71	33
	Mean	36.2	35.3	33.7	38.1	40.0
	SD	5.9	5.9	5.6	6.2	6.1
	Median	35.8	35.5	32.2	38.5	40.0
	IQR	(32.5–40.8)	(29.9–40.2)	(30.8–38.6)	(34.0–42.1)	(35.9–45.2)
	Range	(25.3–47.6)	(24.3–45.2)	(23.8–42.2)	(22.8–55.9)	(29.2–52.7)
SD (HU)	Number	50	48	13	71	33
	Mean	24.9	25.7	23.9	24.8	25.4
	SD	5.8	5.5	3.8	4.6	4.3
	Median	24.9	24.9	23.6	25.9	25.4
	IQR	(21.9–26.6)	(21.5–27.9)	(22.6–26.1)	(22.0–27.7)	(23.0–26.9)
	Range	(14.1–50.6)	(17.3–41.2)	(15.5–29.4)	(5.3–33.9)	(18.1–41.0)

Variable	Statistic	24 M	36 M	48 M	60 M
Volume (cm^3^)	Number	74	70	64	56
	Mean	87.1	93.4	90.6	92.7
	SD	155.9	167.4	176.0	200.2
	Median	49.8	48.9	42.2	33.0
	IQR	(29.1–95.9)	(23.3–109.6)	(18.9–107.9)	(20.5–85.4)
	Range	(4.0–1276.5)	(4.0–1316.3)	(3.1–1306.5)	(3.1–1388.1)
Heigth (cm)	Number	74	70	64	55
	Mean	6.4	6.3	7.2	7.4
	SD	2.4	2.5	9.4	10.8
	Median	6.0	5.6	5.5	5.5
	IQR	(4.5–7.8)	(4.5–7.5)	(4.5–7.5)	(4.5–6.6)
	Range	(3.0–15.6)	(2.5–15.5)	(2.0–78.0)	(3.0–84.0)
Density (HU)	Number	74	70	64	56
	Mean	39.4	40.5	41.0	41.7
	SD	7.6	8.4	8.8	8.8
	Median	39.3	40.4	40.2	41.0
	IQR	(33.5–44.0)	(36.0–45.0)	(34.6–45.5)	(35.6–45.1)
	Range	(25.70–61.80)	(24.30–78.70)	(24.00–78.30)	(25.70–75.60)
SD (HU)	Number	74	70	64	56
	Mean	25.8	26.1	26.2	25.2
	SD	5.3	5.4	4.8	4.6
	Median	25.0	25.0	26.6	25.3
	IQR	(22.6–28.9)	(22.2–29.8)	(22.6–29.4)	(21.9–27.8)
	Range	(16.6–49.0)	(17.6–48.5)	(17.0–37.7)	(14.9–35.5)

*IQR* Interquartile range*, SD* Standard deviation

**Fig. 4:**
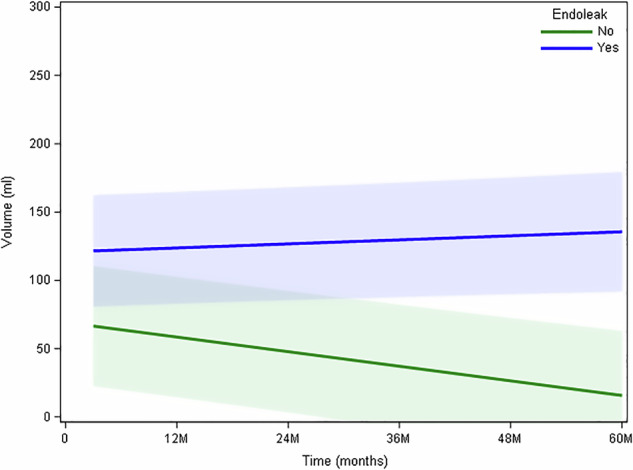
A significant decrease in maximal aneurysm volume over time is observed in patients without endoleak on follow-up scans (green curve, *p* < 0.001). In patients with endoleak on follow-up scans, a slight nonsignificant increase in maximal aneurysm volume is observed (blue curve, *p* = 0.082) (see text for the borderline *p*-value). M, Months

Patients without endoleak on follow-up CT-scan have a significant decrease of the maximal aneurysm sac volume over time with a slope of -0.891 cc/month (95% CI (-1.200 to -0.581), *p* < 0.001. In patients with an endoleak on follow-up CT, without significant evolution in maximal aneurysm sac volume over time with a slope of 0.246 cc/month (95% CI (-0.031–0.523), *p* = 0.082 (Fig. 4).

**Fig. 5:**
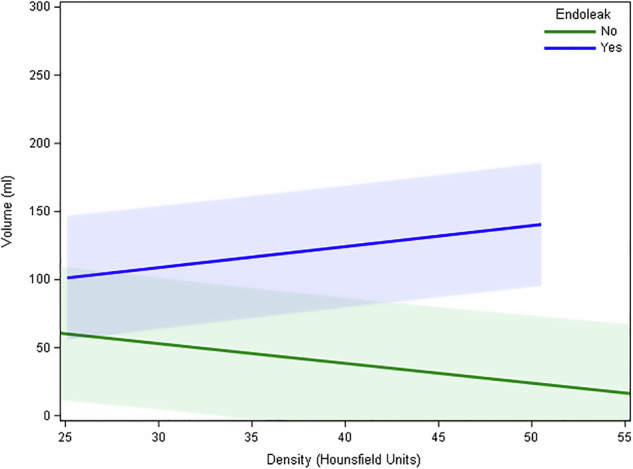
A significant, negative association (*p* < 0.001) between maximal aneurysm volume and thrombus density was revealed in patients without endoleak on follow-up CT scans (green curve); a significant, positive association between maximal aneurysm volume and thrombus density was found in patients with endoleak on follow-up CT scans (*p* < 0.001) (blue curve)

Analysis of the relationship between thrombus density and maximal aneurysm volume over time reveals a significant negative association between aneurysm volume and thrombus density over a slope of -1.450 UH/cc (95% CI (-2.326 to -0.574) and *p* = 0.001; in patients with an endoleak on follow-up CT scans, a significant positive association was found between maximal aneurysm volume and thrombus density over a slope of 1.543 UH/cc (95% CI (0.948–2.138), *p* < 0.001) (Fig. 5).

Intraobserver and interobserver variability data, based on 10 patient imaging datasets are summarised in Table 2, showing values of intraclass correlation coefficient > 0.850.

**Table 2:** Intraobserver repeatability and interobserver reproducibility for measuring maximal aneurysm volume, aneurysm length, and thrombus density

Comparison	ICC (95% confidence interval)
Intraobserver repeatability (Reader 1)	0.943 (0.907–0.965)
Intraobserver repeatability (Reader 2)	0.864 (0.788–0.915)
Interobserver reproducibility	0.868 (0.815–0.907)

*ICC* Intra-class correlation coefficient

## Discussion

This study demonstrates a progressive increase in thrombus density, measured on unenhanced CT, over time, of the excluded aortic aneurysm sac after EVAR, both in patients with and without an underlying endoleak. This finding might be in line with the findings of Cornelissen et al [8] using quantitative analysis of magnetic resonance imaging and demonstrating unorganised thrombus in the excluded aneurysm sac two years after EVAR, irrespective of the presence of additional endoleak or not in the excluded aneurysm sac; in addition, these authors found a pooling of blood after contrast injection in 9 out of 14 cases (64%). This is different to patients presenting with an endoleak on CE-CT: in these cases, a slow and uneven inflow of contrast in the aneurysm sac over time is identified [9].

In our patients with an associated endoleak, the slope of increased thrombus density over time was steeper compared to patients without an associated endoleak. This result seems to be in line with thrombus density measurements in patients with acute, large ischaemic stroke. Fresh, rich red cell thromboembolic material, measured in patients with an acute stroke, had a higher density of the thrombus material, compared to thrombotic material, captured in a stent-retriever or aspiration catheter and presenting with a higher composition of platelets and cellular debris, as found in patients with an acute stroke related to an old thrombotic embolus [10, 11]. Patients with endoleak might have also more red blood cells and/or a fresh, rich red blood cell thrombus in the excluded aneurysm sac, compared to patients without endoleak, with a lower density thrombus in the excluded aneurysm sac, related to an old and more organised thrombotic material, with a higher composition of platelets and cellular debris. Rouer et al [12] did not find a difference in thrombus density one month after EVAR between patients with and without type II endoleak; however, seven months after EVAR, the thrombus density was significantly higher in patients with an additional type II endoleak compared to patients without endoleak, which seems to confirm our data.

The presented study also confirms the results of other studies [7, 8], demonstrating a progressive significant decrease in aneurysm volume in patients without a visible endoleak on follow-up CT after EVAR, compared to patients with a visible endoleak (*p* < 0.001).

Finally, when combining aneurysm sac density and volume data, this study clearly demonstrates a clear negative association between aneurysm volume and thrombus density (negative slope) in patients without visible endoleak on CE-CT, compared to patients with a visible endoleak, presenting with a positive association between aneurysm volume and thrombus density (positive slope). In clinical practice, thrombus density and aneurysm sac volume measurements on unenhanced CT might be an additional tool to avoid or selectively administer iodinated contrast agents during follow-up of patients lately after EVAR [13, 14].

Our study has also some limitations. First, this is a single-centre study, including only patients with CE-CT performed during follow-up; the relatively small sample size may be the reason for the only borderline *p*-value (0.082) for the evolution of maximal aneurysm sac volume over time in patients with endoleak. Second, density measurements were performed only by two radiologists; however, intraobserver and interobserver analysis showed good reproducibility. Third, no distinction was made between different types of endoleaks, no prediction for type I/III endoleak development was calculated and no patients with a late onset of endoleak were included in the study. A robust statistical analysis, potentially differentiating type II from type I/III endoleak should be made with a higher number of patients with type II and type I/III endoleak in order to draw conclusions and might be a good topic for a future study. Fourth, the type of endoleak was not analysed as a parameter, potentially influencing the density of the thrombus and the influence of the type of endograft on thrombus density or aneurysm sac volume was not analysed.

In conclusion, this study showed a progressive increase in thrombus density of the aortic aneurysm sac after EVAR over time. This increase was more pronounced in patients with an underlying endoleak. In addition, a negative and positive association between aneurysm volume and thrombus density was found in patients without and with underlying endoleak, respectively.

## Data Availability

The datasets used during this study are available from the corresponding author on reasonable request.

## Abbreviations

CE-CT: Contrast-enhanced computed tomography
CI: Confidence interval
EVAR: Endovascular aneurysm repair
MRI: Magnetic resonance imaging
